# Nutrition in RMDs: is it really food for thought? Focus on rheumatoid arthritis

**DOI:** 10.1186/s41927-020-0113-4

**Published:** 2020-03-10

**Authors:** Alessia Alunno, Elena Nikiphorou, Elena Philippou, Claire Daien, Dieter Wiek, Marios Kouloumas, Maurizio Cutolo

**Affiliations:** 1 Rheumatology Unit, Department of Medicine, University of Perugia, Piazzale Menghini 1, 06129 Perugia, Italy; 2 Centre for Rheumatic Diseases, School of Immunology and Microbial Sciences, King’s College London, London, UK; 3 Rheumatology Department, King’s College Hospital, London, UK; 4 Department of Life and Health Sciences, School of Sciences and Engineering, University of Nicosia, Nicosia, Cyprus; 5 Department of Rheumatology, Montpellier hospital, France and UMR5535, Institut de Génétique Moléculaire de Montpellier, Montpellier University, Montpellier, France; 6 EULAR PARE, Zurich, Switzerland; 7 Deutsche Rheuma-Liga, Bonn, Germany; 8 Cyprus League Against Rheumatism, Aglantzia, Cyprus; 9 Research Laboratory and Division of Clinical Rheumatology, Department of Internal Medicine, University of Genoa, IRCCS San Martino Polyclinic Hospital, Genoa, Italy

**Keywords:** Rheumatoid arthritis, Mediterranean diet, Immune system

## Abstract

**Background:**

The relationship between food and health is known since the antiquity and in the field of rheumatic and musculoskeletal diseases (RMDs), mainly rheumatoid arthritis (RA), a large number of studies has been published over the last 50 years encompassing different aspects of nutrition. This led to postulate a role of nutrients for both primary prevention of RMDs in the general population and secondary prevention of disease flares and complications in patients with an established RMD.

**Main body of abstract:**

We aimed to summarise and critically discuss current evidence on the role of different nutrients and dietary regimens in RMDs with a focus on RA. Over the last years, some seminal papers proved that some compounds, such as salt, can directly modulate the immune system and large epidemiological studies have been linking dietary patters with the risk to develop RMDs. However, physicians’ knowledge about the role of diet in disease prevention and treatment is often poor and ultimately diet is rarely perceived as a companion of pharmacological treatment.

**Conclusions:**

Based on the currently available evidence, we are not (yet?) in the phase of putting diet on the same level as pharmacological treatment in RMDs and in particular, RA, but future studies will likely shed additional light on this controversial topic and at least might suggest a value as dietary prevention of risk factors.

## Background

The relationship between food and health is known since the antiquity, with the extensively used quote from Hippocrates “Let the food by thy medicine” being the most evident proof. However, as often highlighted, the physicians’ knowledge on diet-disease associations is poor and therefore the potential use of diet as a companion of pharmacological treatment often neglected [1]. This is particularly true in the field of rheumatic and musculoskeletal diseases (RMDs) in particular arthritis, since the increase of published papers on this topic does not seem to result in a parallel increase of awareness nor of dietary counselling in general and rheumatology practice. A simple search on “nutrition AND rheumatic diseases” in PubMed results in over one thousands of hits with the oldest paper, on clinical aspects of nutrition in rheumatic fever, dating back in the late 1940s. Interestingly, of these publications, nearly 900 pertain to rheumatoid arthritis (RA). The concept of nutrition in RMDs goes far beyond the control of weight and encompasses many different aspects. RMDs result from the action of different environmental factors on genetically predisposed individuals, with food being by definition an environmental factor to which every human being is daily exposed. However, it is up to the person how to mix and match the nutrients and if to complement it with voluptuary habits such as alcohol and coffee drinking. This highlights the huge potential to modulate dietary habits for preventive purposes not only in patients with an established RMD as secondary prevention [2] but also in the general population to reduce the risk of RMD development.

## Main body of the article

### ‘Feeding’ RA prevention while ‘starving’ autoimmunity and inflammation

Overall, a ‘western’ type diet might increase the risk of RMDs both directly through increasing inflammation [3] and indirectly through increasing insulin resistance, obesity and associated co-morbidities [4]. On the other hand, as demonstrated by Hu and colleagues in women, a higher adherence to the 2010 Alternative Healthy Eating Index (AHEI-2010) is associated with reduced risk in RA [5]. The AHEI-2010 includes a variety of food/nutrients and defines fruits, vegetables, whole grains, nuts, long-chain n-3 polyunsaturated fatty acids (LC-PUFAs), and moderate alcohol consumption as healthy items, while sugar-sweetened beverages, red/processed meat, trans fat and sodium intake are classified as unhealthy components. While a high consumption of red meat, meat products and overall proteins has been associated with an increased risk of inflammatory polyarthritis [6, 7], the consistent intake of fish, which provides LC-PUFAs displaying anti-inflammatory properties, was linked to decreased risk to develop rheumatoid arthritis (RA) [8] (Table 1). The risk of developing RA is also inversely correlated with olive oil or cooked vegetable consumption [12] and directly correlated with sodium (salt), the latter in a dose-dependent manner [9]. Possible mechanisms include the antioxidant properties of fruit and vegetables, natural tocopherols contained in olive oil acting as radical scavengers, and the pro-inflammatory action of salt. As far as salt is concerned, two recent studies provided the mechanistic link between sodium and autoimmunity by the identification of a salt-sensitive kinase and salt-responsive gut commensals which are involved in the differentiation of pathogenic T helper 17 cells [14, 15]. A reduction in salt intake by RMD patients would thus result in both direct and indirect benefits on the cardiovascular system. In fact, both the immune response and chronic inflammation ultimately participate also in endothelial dysfunction. Other nutrients, such as polyphenols, curcumin or citric acid also display antioxidant properties, while in contrast, saturated fatty acids promote oxidative stress [16]. Other research evidence suggests that sugar-sweetened drinks are associated with an increased risk of seropositive RA [10] and in support of this, consumption of ≥5 times/week of high-fructose corn-syrup sweetened soft drinks, fruit drinks and apple juice has been associated with arthritis in young US adults [17]. This may be linked to the fact that consumption of excess free fructose (defined as fructose to glucose ratio > 1:1) contributes to fructose reactivity in the gastrointestinal tract and intestinal in situ formation of advanced glycation end products, which once absorbed, travel beyond the intestinal boundaries to other tissues and promote inflammation [17]. As far as alcoholic drinks are concerned, surprisingly consumption of 3–5 standard drinks/week has been associated with 22–31% reduced risk of developing RA compared to non-alcoholic drinks [13]. One item not included in the AHEI 2010 but deserving attention due to its widespread use, is coffee. A recent meta-analysis found that consumption of ≥4 cups coffee per day increases risk of seropositive, but not seronegative RA [11]. However, these results should be interpreted with caution since they are in striking contrast with data from in vitro studies demonstrating that caffeine owns anti-inflammatory properties via protein kinase A activation, including reduced production of autoantibodies [18]. Diet, in addition, is one of the main drivers of gut microbiota composition which in turn is closely related to the immune system. An altered microbiota, namely ‘dysbiosis’ can induce gut permeability and pro-inflammatory cells which could favour the development of autoimmunity [19]. The western diet, deprived in dietary fibre further favours dysbiosis and therefore the triggering of pro-inflammatory cells [20].

**Table 1:** Nutrients associated with higher or lower risk to develop rheumatoid arthritis (RA)

Evidence	Food/Drink	Ref
↑ risk to develop RA	-red meat/meat products	[6, 7]
	-salt	[9]
	-sugar-sweetened drinks	[10]
	-coffee (≥4 cups/day)	[11]
↓ risk to develop RA	-fish	[8]
	-olive oil	[12]
	-cooked vegetables	[9]
	-alcoholic drinks (3–5 drinks/week)	[13]

### Is diet a possible concomitant DMARD in RA?

The level of evidence in clinical studies on diet manipulation in patients with RMDs is low, since good-quality trials are difficult to conduct. Restrictive-diet studies often lack appropriate control groups since it is not possible to have a placebo group and since the placebo effect of diet changes is expected to be important, any firm conclusion cannot be drawn. This is the case for two open studies on gluten and vegan free diets, which suggest a slight improvement of disease activity [21, 22]. However, some data are suggesting that a gluten-free diet increases cardiovascular risk factors [23] and might increase cardiovascular events [24]. This is a major point as patients with RMDs already have an increased risk of cardiovascular events [25]. No pre-clinic or clinical study is available examining a lactose-free diet. However, a lactose-free diet favors calcium deficiency which can promote osteoporosis in patients that are already at increased risk [26]. Many open studies have assessed the impact of fasting on RA activity. The fasting protocols strongly vary between studies and a beneficial effect on pain, morning stiffness and inflammation has been observed, however, this effect is transient, as inflammation relapse with food resumption [27, 28]. Open studies suggested a small but significant effect of the Mediterranean diet on pain and global assessment [29]. Stronger evidence shows that the Mediterranean diet decreases cardiovascular events and cardiovascular mortality [30]. This is of particular relevance due to the well-established increased cardiovascular risk in rheumatic diseases [31]. The Mediterranean diet is enriched in fiber, olive oil, a source of mono-unsaturated fatty acid and in fish, an important source of dietary n-3 PUFA. The effect of n-3 PUFA supplements on RA activity has been assessed in several placebo-controlled studies. A very recent meta-analysis of randomized controlled trials assessing the effect of n-3 PUFA supplementation showed a slight but significant decrease of pain, tender joints, health assessment questionnaire and erythrocyte sedimentation rate [32]. Consistent with those results, a randomized controlled trial showed that the consumption of 10 ml per day of fish oil doubles the chance of achieving ACR remission in patients with early RA treated with a combination of conventional synthetic disease-modifying anti-rheumatic drugs (csDMARDs) [33]. Interim data from the total management of risk factors in rheumatoid arthritis patients to lower morbidity and mortality (TOMORROW) study revealed that daily consumption of monounsaturated to saturated fatty acids (MUFA/SFA) as part of a Mediterranean diet is inversely correlated with disease activity while high MUFA intake is an independent predictor of remission in the RA [34]. On the contrary, probiotic use in RA showed controversial results, which could be explained by the use of different bacterial strains [35]. A recent randomized placebo-controlled trial showed that the association of *Lactobacillus acidophilus*, *Lactobacillus casei* and *Bifidobacterium bifidum* probiotics with fiber supplement could significantly decrease disease activity [36].

### What can we learn from existing evidence?

Since people do not consume individual foods but diets, the pursuit of RMD/arthritis prevention in the general population and RMD control in patients is ultimately a matter of dietary patterns. Adherence to the Mediterranean diet is generally recommended, with a plant-based regimen abundant in wholegrains, legumes, five or more fruit and vegetables per day, preferably seasonal. Daily consumption of extra-virgin olive oil, 1–2/week consumption of fatty fish (sardines, salmon, seabeam, seabass and trout), weekly consumption of other types of fish and poultry and limited consumption of red meat to 1–2 per month encompasses all the current available evidence. It is also recommended to avoid or reduce consumption of sugar-sweetened drinks and salt, limit coffee consumption to 3 cups a day, and if alcohol is consumed, drink in moderation and with meals. This latter concept also fits with the general recommendation to reduce, ideally avoid, alcohol consumption by people receiving potentially hepatotoxic DMARDs [37]. Restrictive diets should not be recommended to patients with arthritis, as no evidence supports benefits and data suggests risk. Modulation of microbiota through specific multistrain probiotics and fibers might be a promising strategy but to date only low quality evidence is available. Finally, food and drink intake should be balanced with requirements by controlling portion sizes and engaging in daily physical activity aiming to maintain a normal body weight, as recently underscored also by EULAR recommendations on physical activity in RMDs [38], is imperative.

## Conclusions

In conclusion, the currently available evidence disputes, at least in part, Hippocrates’ notion that food should be a medicine by definition. We are not (yet?) in the phase of putting diet on the same level as pharmacological treatment in RMDs and in particular arthritis, but we are confident that future studies will shed additional light on this controversial topic and at least might suggest a value as dietary prevention of risk factors. However, although Greeks and Romans were the first populations following the Mediterranean diet, Hippocrates recognised himself that the definition of food as medicine should be performed with caution, and anticipating centuries of research, eventually concluded: “if we could give every individual the right amount of nourishment and exercise, not too little and not too much, we would have the safest way to health.” The Romans added a wise sentence: “*Est modus in rebus*” that doctors should always suggest to their patients and to themselves regarding the pleasures of the table!

## Data Availability

Not applicable.

## Abbreviations

AHEI-2010: 2010 Alternative Healthy Eating Index
csDMARDs: Conventional synthetic disease-modifying anti-rheumatic drugs
LC-PUFAs: Long-chain n-3 polyunsaturated fatty acids
MUFA/SFA: Monounsaturated to saturated fatty acids
RA: Rheumatoid arthritis
RMDs: Rheumatic and musculoskeletal diseases
TOMORROW: Total management of risk factors in rheumatoid arthritis patients to lower morbidity and mortality

## References

[1] Smith R (2004). Let food be thy medicine …. BMJ.

[2] Cutolo M, Nikiphorou E (2018). Don’t neglect nutrition in rheumatoid arthritis!. RMD Open.

[3] Minihane AM, Vinoy S, Russell WR, Baka A, Roche HM, Tuohy KM (2015). Low-grade inflammation, diet composition and health: current research evidence and its translation. Br J Nutr.

[4] Qin B, Yang M, Fu H, Ma N, Wei T, Tang Q (2015). Body mass index and the risk of rheumatoid arthritis: a systematic review and dose-response meta-analysis. Arthritis ResTher.

[5] Hu Y, Sparks JA, Malspeis S (2017). Long-term dietary quality and risk of developing rheumatoid arthritis in women. Ann Rheum Dis.

[6] Pattison DJ, Silman AJ, Goodson NJ, Lunt M, Bunn D, Luben R (2004). Vitamin C and the risk of developing inflammatory polyarthritis: prospective nested case-control study. Ann.Rheum.Dis.

[7] Grant WB (2000). The role of meat in the expression of rheumatoid arthritis. Br J Nutr.

[8] Di GD, Wallin A, Bottai M, Askling J, Wolk A (2014). Long-term intake of dietary long-chain n-3 polyunsaturated fatty acids and risk of rheumatoid arthritis: a prospective cohort study of women. Ann.Rheum.Dis.

[9] Salgado E, Bes-Rastrollo M, de Irala J, Carmona L, Gomez-Reino JJ (2015). High sodium intake is associated with self-reported rheumatoid arthritis: a cross sectional and case control analysis within the SUN cohort. Medicine (Baltimore).

[10] Hu Y, Costenbader KH, Gao X, Al-Daabil M, Sparks JA, Solomon DH (2014). Sugar-sweetened soda consumption and risk of developing rheumatoid arthritis in women. Am J Clin Nutr.

[11] Lee YH, Bae SC, Song GG (2014). Coffee or tea consumption and the risk of rheumatoid arthritis: a meta-analysis. Clin Rheumatol.

[12] Linos A, Kaklamani VG, Kaklamani E, Koumantaki Y, Giziaki E, Papazoglou S (1999). Dietary factors in relation to rheumatoid arthritis: a role for olive oil and cooked vegetables?. Am J Clin Nutr.

[13] Lu B, Solomon DH, Costenbader KH, Karlson EW (2014). Alcohol consumption and risk of incident rheumatoid arthritis in women: a prospective study. Arthritis Rheum.

[14] Wu C, Yosef N, Thalhamer T, Zhu C, Xiao S, Kishi Y (2013). Induction of pathogenic TH17 cells by inducible salt-sensing kinase SGK1. Nature.

[15] Wilck N, Matus MG, Kearney SM, Olesen SW, Forslund K, Bartolomaeus H (2017). Salt-responsive gut commensal modulates TH17 axis and disease. Nature.

[16] Mateen S, Moin S, Zafar A, Khan AQ (2016). Redox signaling in rheumatoid arthritis and the preventive role of polyphenols. Clin Chim Acta.

[17] DeChristopher LR, Uribarri J, Tucker KL (2016). Intake of high-fructose corn syrup sweetened soft drinks, fruit drinks and apple juice is associated with prevalent arthritis in US adults, aged 20-30 years. Nutr Diabetes.

[18] Dahan S, Segal Y, Shoenfeld Y (2017). Dietary factors in rheumatic autoimmune diseases: a recipe for therapy?. Nat Rev Rheumatol.

[19] Jubair WK, Hendrickson JD, Severs EL, Schulz HM, Adhikari S, Ir D (2018). Modulation of inflammatory arthritis by gut microbiota through mucosal inflammation and autoantibody generation. Arthritis Rheum.

[20] Daïen CI, Pinget GV, Tan JK, Macia L (2017). Detrimental impact of microbiota-accessible carbohydrate-deprived diet on gut and immune homeostasis: an overview. Front Immunol.

[21] Elkan A-C, Sjöberg B, Kolsrud B, Ringertz B, Hafström I, Frostegård J (2008). Gluten-free vegan diet induces decreased LDL and oxidized LDL levels and raised atheroprotective natural antibodies against phosphorylcholine in patients with rheumatoid arthritis: a randomized study. Arthritis Res Ther.

[22] Hafström I, Ringertz B, Spångberg A, von Zweigbergk L, Brannemark S, Nylander I (2001). A vegan diet free of gluten improves the signs and symptoms of rheumatoid arthritis: the effects on arthritis correlate with a reduction in antibodies to food antigens. Rheumatology (Oxford).

[23] Potter MDE, Brienesse SC, Walker MM, Boyle A, Talley NJ (2018). Effect of the gluten-free diet on cardiovascular risk factors in patients with coeliac disease: A systematic review. J Gastroenterol Hepatol.

[24] Lebwohl B, Cao Y, Zong G, Hu FB, Green PHR, Neugut AI (2017). Long term gluten consumption in adults without celiac disease and risk of coronary heart disease: prospective cohort study. BMJ.

[25] Bengtsson K, Forsblad-d’Elia H, Lie E, Klingberg E, Dehlin M, Exarchou S (2017). Are ankylosing spondylitis, psoriatic arthritis and undifferentiated spondyloarthritis associated with an increased risk of cardiovascular events? A prospective nationwide population-based cohort study. Arthritis Res Ther.

[26] Haugeberg G, Uhlig T, Falch JA, Halse JI, Kvien TK (2000). Bone mineral density and frequency of osteoporosis in female patients with rheumatoid arthritis: results from 394 patients in the Oslo County rheumatoid arthritis register. Arthritis Rheum.

[27] Müller H, de Toledo FW, Resch KL (2001). Fasting followed by vegetarian diet in patients with rheumatoid arthritis: a systematic review. Scand J Rheumatol.

[28] Udén AM, Trang L, Venizelos N, Palmblad J (1983). Neutrophil functions and clinical performance after total fasting in patients with rheumatoid arthritis. Ann Rheum Dis.

[29] McKellar G, Morrison E, McEntegart A, Hampson R, Tierney A, Mackle G (2007). A pilot study of a Mediterranean-type diet intervention in female patients with rheumatoid arthritis living in areas of social deprivation in Glasgow. Ann Rheum Dis.

[30] Guasch-Ferré M, Hu FB, Martínez-González MA, Fitó M, Bulló M, Estruch R (2014). Olive oil intake and risk of cardiovascular disease and mortality in the PREDIMED study. BMC Med.

[31] Mackey RH, Kuller LH, Moreland LW (2018). Update on cardiovascular disease risk in patients with rheumatic diseases. Rheum Dis Clin N Am.

[32] Gioxari A, Kaliora AC, Marantidou F, Panagiotakos DP (2018). Intake of ω-3 polyunsaturated fatty acids in patients with rheumatoid arthritis: A systematic review and meta-analysis. Nutrition.

[33] Jung SM, Min HK, Koh JH (2014). Salt aggravates arthritis by Th17 polarization. Arthritis Rheum.

[34] Matsumoto Y, Sugioka Y, Tada M (2018). Monounsaturated fatty acids might be key factors in the Mediterranean diet that suppress rheumatoid arthritis disease activity: the TOMORROW study. Clin Nutr.

[35] Mohammed AT, Khattab M, Ahmed AM, Turk T, Sakr N, Khalil MA (2017). The therapeutic effect of probiotics on rheumatoid arthritis: a systematic review and meta-analysis of randomized control trials. Clin Rheumatol.

[36] Zamani B, Farshbaf S, Golkar HR, Bahmani F, Asemi Z (2017). Synbiotic supplementation and the effects on clinical and metabolic responses in patients with rheumatoid arthritis: a randomised, double-blind, placebo-controlled trial. Br J Nutr.

[37] Price S, James C, Deighton C (2010). Methotrexate use and alcohol. Clin Exp Rheumatol.

[38] Rausch Osthoff A, Niedermann K, Braun J (2018). 2018 EULAR recommendations for physical activity in people with inflammatory arthritis and osteoarthritis. Ann Rheum Dis.

